# Corrective bandages and daily manipulations for treatment of congenital vertical talus: a thirteen year follow-up

**DOI:** 10.1007/s00264-022-05685-7

**Published:** 2023-01-11

**Authors:** Elia Utrilla-Rodríguez, Nieves Díaz-Ávila, Antonia Sáez-Díaz, Pedro V. Munuera-Martínez, Manuel Albornoz-Cabello

**Affiliations:** 1 Paediatric Rehabilitation Service, University Hospital “Virgen de Macarena”, Seville, Spain; 2 Axioma Comunicaciones SL, Seville, Spain; 3 Faculty of Nursing, Physiotherapy and Podiatry, University of Seville, Calle Avicena, S/N. 41009, Seville, Spain

**Keywords:** Newborn, Congenital vertical talus, Bandages, Conservative treatment

## Abstract

**Purpose:**

To analyze the results of a conservative method for treating congenital vertical talus in children with early start and to know in which cases surgical treatment was needed.

**Methods:**

A retrospective analysis of all children diagnosed with idiopathic vertical talus was carried out during the years 2008–2021. Thirty-two children (46 feet) were finally included. Children were treated with serial manipulations, muscle stimulation, and corrective bandages. Age at the time of initiation of treatment, duration of treatment, and correction or not of the deformity without surgical intervention were recorded as variables of interest. The talocalcaneal angle, TAMBA, and ankle range of motion were measured before treatment, after treatment, and at the end of the follow-up period. Statistics decision tree was used to determine which variable best discriminated whether the patient needed surgery. To complement the tree diagram, a two-step cluster analysis was carried out.

**Results:**

After treatment, TAMBA and talocalcaneal angle changed from “vertical” to “oblique” category in 45 and 37 feet, respectively. The pathological dorsal flexion of the ankle changed to normal in 37 feet and ankle plantar flexion was normal in 46 feet. These variables showed significant changes between the three measurement moments. The results of the statistics decision tree and cluster analysis indicate that “No surgery” was associated with an age equal to or lower than one week when treatment was started, and with an ankle plantar flexion range of motion lower than 36°.

**Conclusions:**

The beginning of this conservative treatment in the first week of life and having a plantar flexion of the ankle lower than 36° were related to the success of the treatment without surgery.

## Introduction

Congenital vertical talus (CVT) is a rare foot deformity, occurring in about 1 in 10,000 [1] to 1 in 150,000 births [2], in which there is a rigid and irreducible dorsal dislocation of the navicular bone on the talar head, with the talus fixed vertically, the hindfoot equinus, and the forefoot dorsiflexed [3–8]. The aetiology is unknown in approximately 50% of cases; the remaining 50% being associated with neuromuscular disorders or other chromosomal abnormalities [9, 10].

**Fig. 1:**
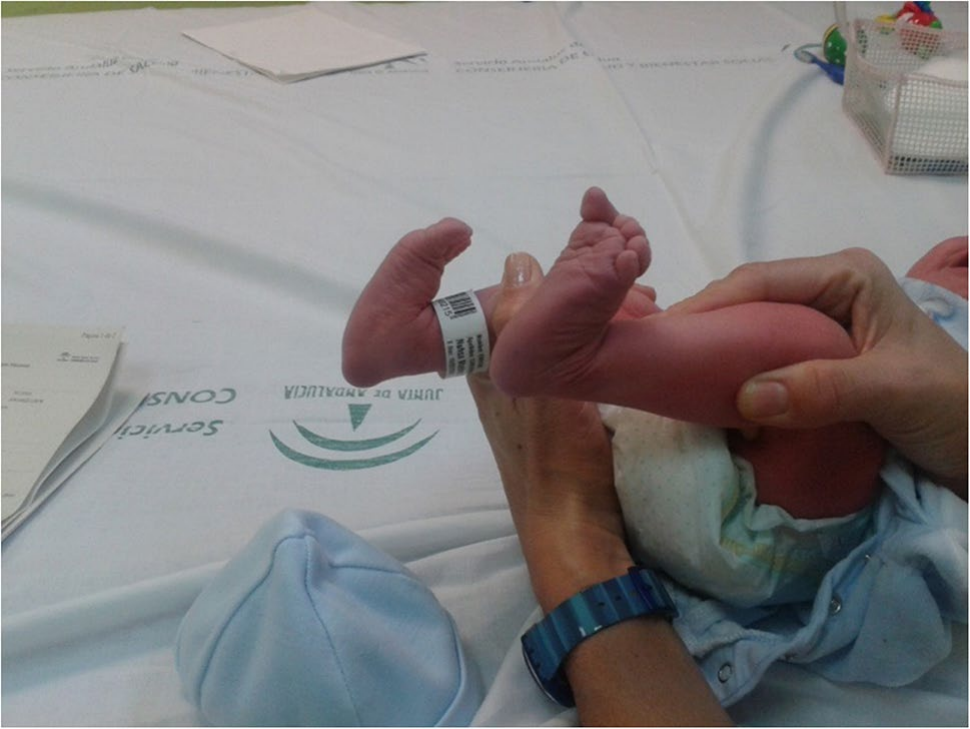
Newborn with bilateral congenital vertical talus

The clinical appearance is a rocker-bottom deformity, abduction of the forefoot, and dorsiflexion of the midfoot, with stiffness and equinus in the hindfoot [1], as well as hypermobility between the midfoot and the hindfoot (Fig. 1). The head of the talus is palpable on the plantar medial aspect of the midfoot with contracture of dorsal structures and tissues [6, 7, 11–14]. Untreated children can develop foot deformities with weight-bearing on the talus resulting in painful callosities, aggravated hypermobility between the midfoot and the hindfoot, weak push-off, and rocker-bottom feet [15].

CVT treatment is commonly surgical and involves extensive soft tissue releases to allow restoration of the normal anatomical relationships between the bones of the foot [14]. A high rate of complications may appear after surgery, such as the risk of undercorrection, overcorrection, or recurrence, joint stiffness, avascular necrosis of the talus, or loss of functionality, which could require additional surgery and increase the risk of morbidity. Less invasive methods have shown excellent results [16]. Extensive open surgical procedures are associated with a high rate of complications [17], but complications can appear even in procedures that involve only soft tissue release [15].

The development of effective conservative therapeutic procedures has allowed improved correction and lower rates of surgical intervention, avoiding or reducing the potential long-term complications of surgical treatment [3, 4, 6, 7, 18, 19]. A conservative treatment for CVT consisting of daily manipulation and corrective bandages has shown effectiveness in clubfoot [20], and metatarsus varus [21], especially when it began in the first weeks of life. However, the results of this conservative protocol to treat CVT have not previously been evaluated related to the age at the start of treatment. This study aims to retrospectively analyze the results of a conservative method of treating CVT in children with early start and to know in which cases surgical treatment was needed.

## Materials and methods

### Design

A retrospective study was designed with all children born with CVT at the Virgen Macarena Hospital in Seville during the years 2008–2021 who were treated with corrective bandaging. This study was approved by the Ethics Committee of Virgen Macarena University Hospital.

### Participants

The selection criteria included having been diagnosed with idiopathic vertical talus or group V according to Hamanishi classification [5], with lateral TAMBA ≥ 30°, and not having received previous treatment, conservative or surgical. Patients who had other congenital syndromes or other associated congenital diseases, along with those children whose medical histories were incomplete or had not finished treatment, were excluded from the study.

### Intervention

The children were treated with serial manipulations, muscle stimulation, and corrective bandages. Once clinical correction was achieved, a foot and ankle orthosis (AFO) was applied at night for one year.

Treatment sessions were held three days a week for 20 min per foot and consisted of a first phase of stretching the retracted structures of the foot by corrective manipulations in plantar flexion and inversion while applying pressure to the medial surface of the head of the talus, avoiding the shortening of the peroneal muscles. Alterations of the deformity in all planes were corrected simultaneously.

**Fig. 2:**
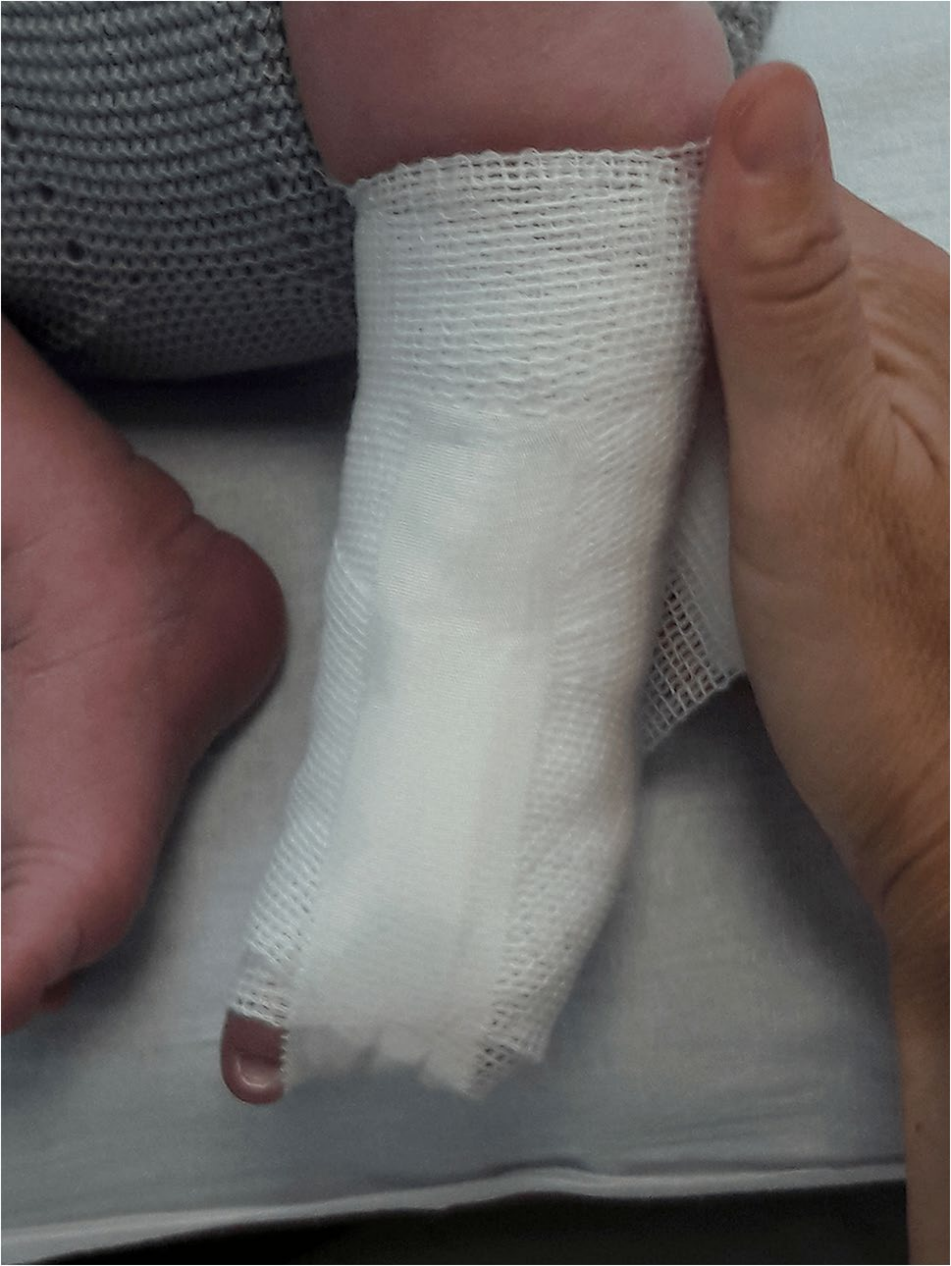
Adhesive tape from the front of the leg, passing through the toes, and fixed on the back of the leg, keeping the foot plantarflexed to ensure adequate stretching of the retracted dorsolateral tendons, the joint capsules, and the skin

To maintain the correction obtained by manipulation, a bandage was applied: First, a circular cotton bandage was placed as cushioning; then, a slightly tighter bandage was wrapped, which slightly corrected the dorsiflexion component; next, an adhesive tape was applied that extended from the anterior surface of the leg, passing through the toes, to be fixed on the posterior aspect of the leg, to keep the foot plantarflexed (Fig. 2); lastly, a final layer of bandage was applied to prevent the adhesive tape from unraveling. This bandage was renewed until the clinical correction of the deformity was confirmed. Manipulations were the same before applying each bandage.

The children’s parents were taught foot exercises to maintain the correction achieved. The use of ankle–foot orthoses was recommended for this purpose, only when clinical correction was not achieved in three weeks of corrective bandages treatment.

Each patient was treated by the same physical therapist for each session. The evaluations of pretreatment clinical and radiological measurements, one year after treatment, and the latest measurements at the end of the follow-up period when clinical correction was achieved, were carried out by the same observer (a different therapist).

### Outcome measures

The present study analyzes the follow-up of CVT feet in children on a scheduled basis and covers 2008 to 2021, with the last measurement in 2021 (the average follow-up of the feet was 4 years).

**Fig. 3:**
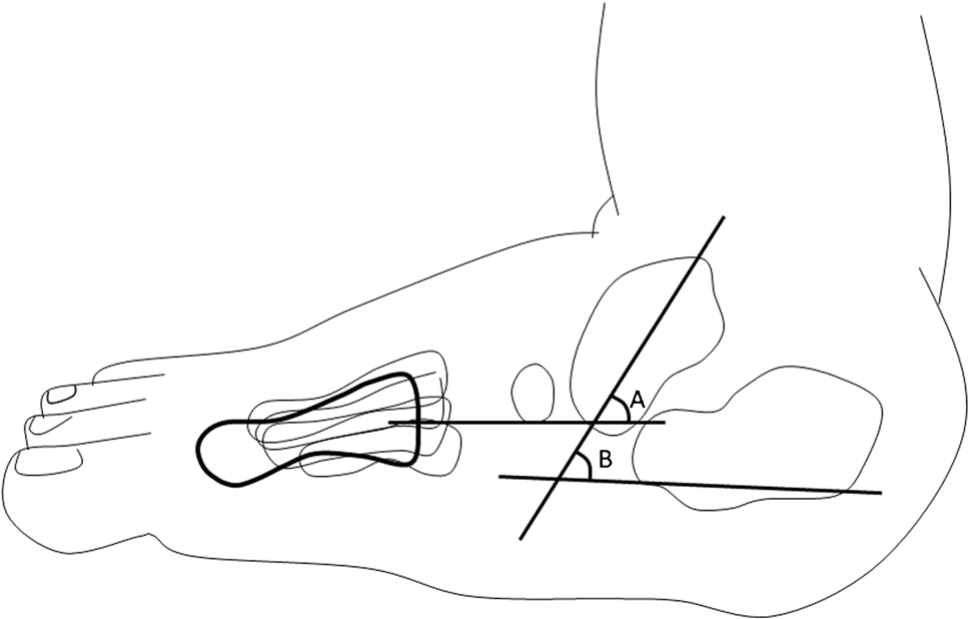
Simple line diagram of the TAMBA (**A**) and the talocalcaneal angle (**B**)

**Fig. 4:**
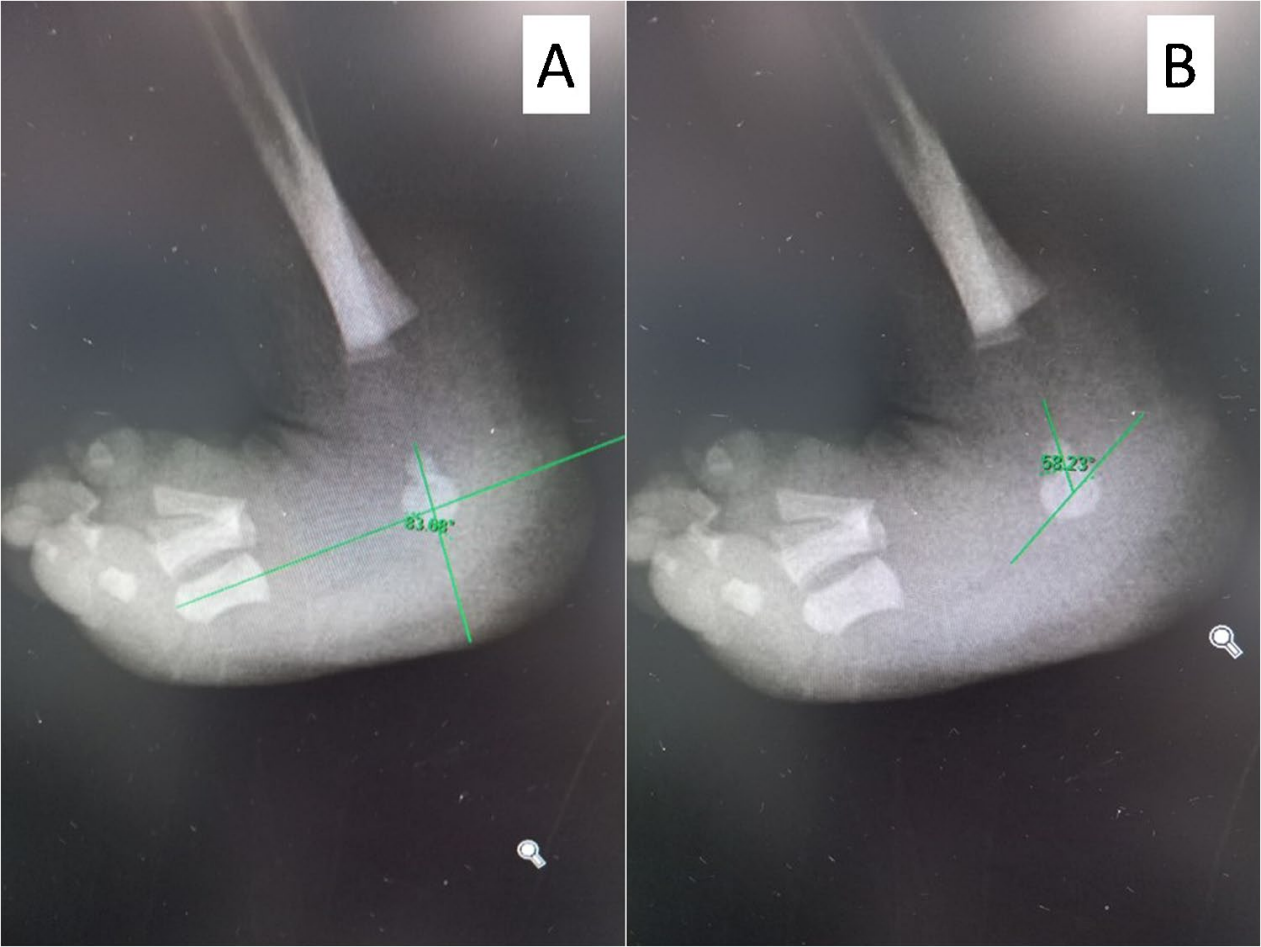
Xray outlining the angles measured, TAMBA (**A**) and the talocalcaneal angle (**B**)

The medical history and radiographs for each patient were reviewed. The age at the time of the initiation of treatment, duration of conservative treatment, and correction or not of the deformity without surgical intervention were recorded. In the lateral X-rays, the talocalcaneal angle (TCA) and the talar axis first-metatarsal base angle (TAMBA) were measured (Figs. 3 and 4). These angles were measured three times: (1) before the intervention, (2) one year after the intervention, and (3) at the last follow-up after clinical and radiological correction. The position of the feet and the radiographic landmarks were used as described by Becker-Andersen and Reiniann [20]. Passive ankle plantar flexion and dorsal flexion were measured by a single examiner with a handheld goniometer three times, in order to use the mean value for statistical analysis. In addition, the American Orthopaedic Foot and Ankle Society scale was obtained as a functional score measurement at the end of the follow-up period.

### Statistical analysis

Data were analyzed using SPSS v.25 (SPSS Science, Chicago, USA). Statistical analysis included a general descriptive analysis. For the analysis of the three measurements (before the treatment, 1 year after the treatment, and at the last follow-up) of the variables TAMBA, TCA, ankle dorsal flexion and plantar flexion over time, the normality of the data was checked using the Shapiro–Wilk test and the homogeneity of the variances was tested using Levene’s test. Since the existence of a good adaptation of residuals to normality could not be confirmed, the Friedman test was applied, applying the Wilcoxon signed-rank or Student’s *T* test for dependent means as post hoc tests. The effect size was calculated using Rosenthal’s *r* or Cohen’s *d* to analyze the magnitude of the differences. Differences were classified according to the following for both parameters: below 0.2, no effect; 0.2–0.5, small effect; 0.5–0.8, medium effect; above 0.8, large effect.

Statistics decision tree was used as a decision support tool to determine which variable (TAMBA, TCA, ankle dorsal flexion, and ankle plantar flexion) best discriminates whether the patient needs surgery. To complement this tree diagram, a two-step cluster analysis was performed. In the first step, a selection process was carried out in which different groups were formed maintaining the maximum similarity within the groups while minimizing the similarity between them. In the second phase, the classification made in the first step was scored (good, greater than 0.5; sufficient, between 0.2 and 0.5; or poor, lower than 0.2) using the silhouette measure of cohesion and separation coefficient, which measures the quality of the groups formed. The groups were considered valid when the silhouette measure of cohesion and separation coefficient was greater than 0.5. For all analyses, the confidence level a priori was 95%, so experimental *p*-values lower than 0.05 were considered statistically significant.

## Results

Thirty-two children (15 boys and 17 girls, 46 feet) were included in the study. Patients were born with an average weight of 3.14 ± 0.67 kg and a mean height of 48.3 ± 2.50 cm. Twenty-three children (32 feet) were diagnosed, and their conservative treatment started, the first week after birth, and nine (14 feet) after one week (2 within the first month after birth and seven after the first year). Both feet were affected in 14 patients and only one foot in 18.

Fourteen children (43.8%) required two weeks of treatment with daily manipulation and corrective bandages until clinical correction, and 18 (56.2%) required three weeks of intervention. A total of 22 patients (69%) needed an ankle–foot orthosis after three weeks of corrective bandages, and ten patients (31%) did not. Among the children who needed splints, 8 (36.4%) used them for two months, 12 (54.5%) for three months, and only two (9.1%) for four months.

Changes of the study variable categories from the beginning of the treatment until the end of the follow-up can be observed in Table 1. Table 2 shows that quantitative values of TAMBA, TCA, ankle dorsal flexion, and ankle plantar flexion improved significantly, except ankle dorsal flexion from the end of treatment to the follow-up visit. Data related to AOFAS scale at the end of the follow-up period are shown in Table 3. All patients reported more than 75 points.

**Table 1:** Changes in categories of TAMBA, TCA, Ankle dorsal flexion, and ankle plantar flexion over time

	Before the intervention	One year after the intervention	At the end of follow-up after clinical and radiological correction
TAMBA			
Vertical (> 36°)	45	0	0
Oblique (16–36°)	1	46	14
Normal (< 16°)	0	0	12
TCA			
Vertical (> 50°)	42	2	0
Oblique (45–50°)	3	7	1
Normal (< 45°)	1	37	45
Ankle dorsal flexion			
Pathological (> 30°)	40	9	9
Normal (20–30°)	6	37	37
Ankle plantar flexion			
Pathological (< 30°)	6	0	6
Normal (30–50°)	40	46	40

**Table 2:**
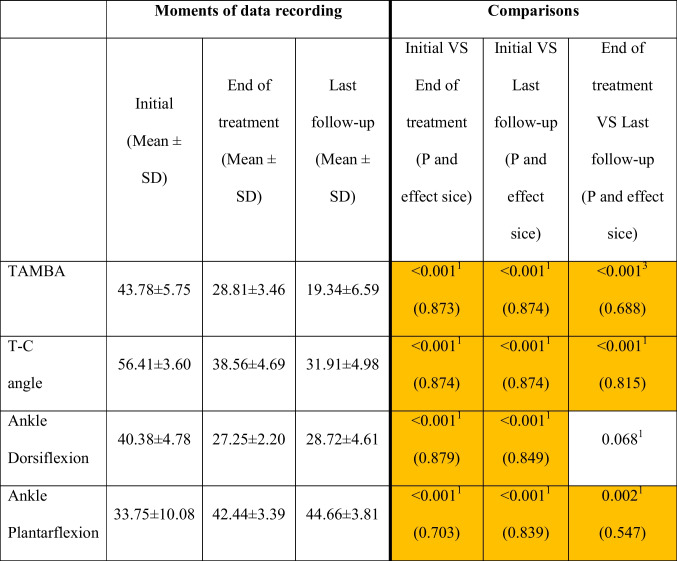
Comparison of the study variables over the 3 moments of data collection

All measurement units were degrees

Mean ± standard deviation (effect size)

^1^Wilcoxon signed-rank test for related samples

^2^Friedman 2-way analysis of variance for related samples

^3^* T*-test for related samples

**Table 3:** AOFAS scores for all patients at the end of the follow-up period

No. of patients	AOFAS score
19	100
3	97
2	94
2	92
1	88
1	86
1	84
2	75
1	73

**Fig. 5:**
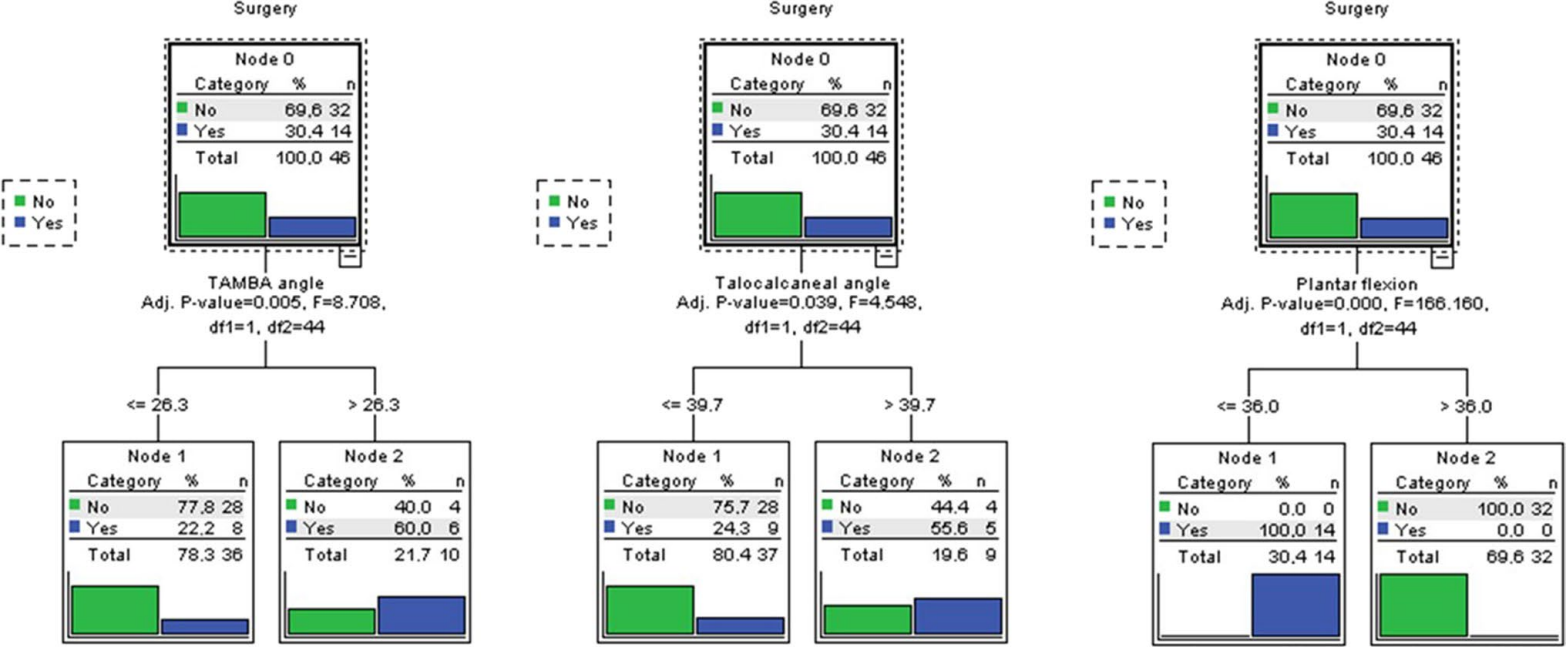
Statistics decision tree identifying groups of patients showing nodes, *F*-statistic values, freedom degrees, and *p*-values. For the TAMBA, the optimum classification value was 26.3°. Patients with a TAMBA angle value lower than or equal to 26.3 were in node 1 (36 feet). This node showed that 77.8% of the patients (28 out of 36) had not been operated on. In node 2 were patients with TAMBA greater than 26.3 (60% were operated, 6/10). Since lower TAMBA values had better follow-up results, we could say that non-operated patients had a higher probability of obtaining a follow-up result lower than 26.3° (*F*_1, 44_ = 8.708, *p* = 0.005). The overall correct percentage of classification was 73.9%. For the TCA, the optimum classification value was 39.7°. 75.7% of the patients (28/37) who showed a TCA equal to or lower than 39.7° (node 1) were not operated (37 feet). Node 2 included feet with a TCA greater than 39.7°, of which 55.6% (5/9) were operated. The lower the TCA, the better the results, so non-operated feet had the highest probability of getting the TCA equal to or lower than 39.7° (*F*_1, 44_ = 4.548, *p* = 0.039). The overall correct percentage of classification was 71.7%. Regarding plantar flexion of the ankle, the best value that classified this variable was 36°. Nodes 1 and 2 correctly classify 100% of cases. All feet that were surgically treated belonged to the group with 36° or less. Otherwise, when the plantar flexion of the ankle is greater than 36°, all feet were not operated. The higher the plantar flexion of the ankle, the better the results, so the best classified cases were the non-operated feet (*F*_1, 44_ = 166.160, *p* ≤ 0.001). The overall correct percentage of classification was 100%

The nominal variable “surgery” was used as the dependent variable in the statistics decision tree, and the variables TAMBA, TAC, and ankle plantar flexion as independent variables. The quick unbiased efficient statistical tree algorithm (QUEST) was employed because the dependent variable was nominal. Figure 5 shows the *F*-statistic value, freedom degrees, and *p*-values. As can be observed, initially (node 0) 69.9% of feet were not operated (32/46), compared to 30.4% that did (14/46).

**Fig. 6:**
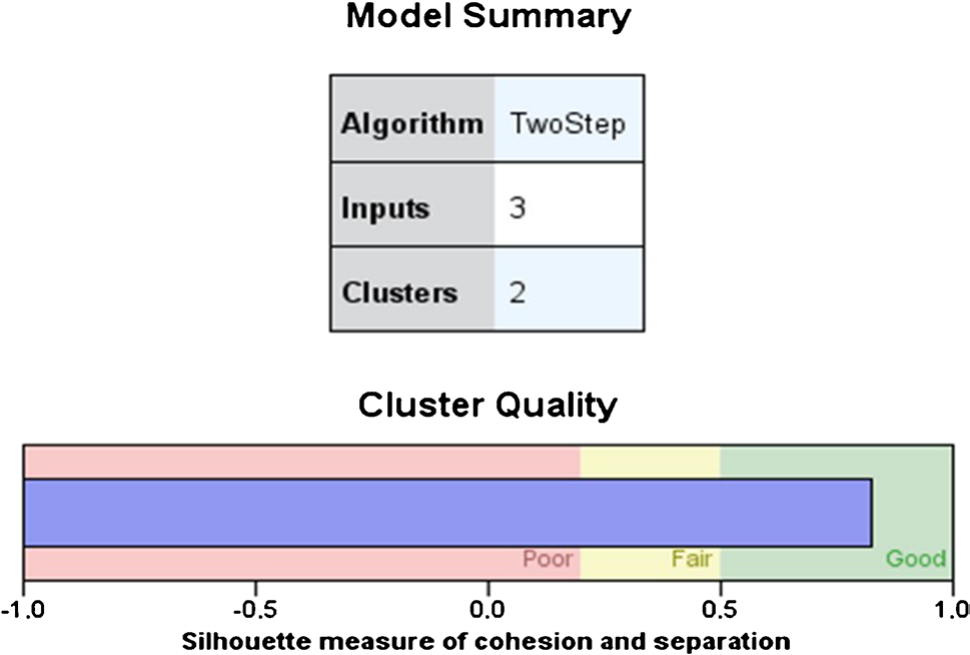
Summary and quality of the cluster analysis model. A silhouette measure of cohesion and separation coefficient of 1 means that all cases are in their cluster centres. A value of − 1 means that all cases are in the centers of other clusters to which they do not belong. A value of 0 means that, on average, the cases are equidistant between the center of their own conglomerate and the center of another nearby conglomerate. Therefore, a result in the “Good” zone means that the data strongly evidence the structure of the conglomerates. A result in the “Sufficient” area means that the data show this cluster structure in a less evident way, and a result in the “Poor” area reflects that the data does not provide significant evidence of the cluster structure

Ankle plantar flexion was the variable chosen to perform the two-step cluster analysis. The silhouette measure of cohesion and separation coefficient was 0.73 (Fig. 6). Two groups were classified according to whether they need surgery or not, using the “plantar flexion of the ankle” and “start of treatment” variables (Table 4).

**Table 4:** Values of the age at start of treatment and range of ankle plantar flexion used in assembling the conglomerates for predicting the need of surgery. “No surgery” was associated with an age equal to or less than 1 week when treatment was started, and with a plantar flexion of the ankle less than 36°. “Surgery” was associated with an age equal to or greater than 4 years, and a plantar flexion of the ankle greater than 40°. An uncertainty area was found when the range of plantar flexion of the ankle was between 35 and 40°

	Age at start of treatment	Ankle plantar flexion range of motion
No surgery	< = 7 days	< 36°
Interval of uncertainty		36–40°
Surgery	> 1457 days (4 years)	> 40°

## Discussion

The present study describes a treatment method based on the application of corrective bandages performed on 46 feet with CVT. To the authors’ knowledge, this is the first study to report the results of a conservative method for CVT treatment using corrective bandages. Furthermore, to our knowledge, this study reports the outcome in the largest group of patients in the published literature.

Other authors have reported positive results with conservative treatment in isolated cases of CVT [1]. A critical review addressed the efficacy of conservative treatments for the flexible flat foot in children [2]. Conservative treatment of the flat foot in children has mainly involved in-shoe foot orthoses and orthopaedic footwear for the flexible flat foot. Exercise for flat feet has also been described, but in the form of barefoot time and prescribed activities [2, 8].

Historically, CVT correction was surgical, with soft tissue releases performed to restore normal anatomical relationships between the bony structures of the foot. However, surgical treatment has been commonly associated with stiffness, high recurrence rate, chronic pain, and wound problems [8, 14]. In the 1970s, techniques for the effective reduction of the talonavicular joint were described, later developing different surgical techniques in which extensive soft tissue release was performed in one or two stages [20]. The first stage consists of lengthening the contracted tendons, releasing the associated capsular contractures, and reducing the talonavicular and subtalar joint complex. The second stage consists of lengthening the Achilles tendon and the peroneal tendons. However, the complications associated with both techniques, such as avascular necrosis of the talus, under or overcorrection of the deformities, and possible recurrences, are concerning [7, 13, 16]. Long-term problems include severe stiffness of the ankle and subtalar joint and the development of degenerative arthritis, a high recurrence rate, chronic pain, and wound problems [3, 14, 16].

Less invasive techniques have been associated with better results. Dobbs et al. [7] proposed a treatment method similar to the conservative treatment method applied by Ponseti for congenital clubfoot. They performed foot manipulations and placement of corrective casts, followed by securing the talonavicular joint and percutaneous tenotomy of the Achilles tendon in 19 feet. Although they showed good results in clinical appearance and functionality, among the 19 feet treated, six had recurrences after two years. Aslani [3] applied the Dobbs technique to 15 feet and Bhaskar to 4 [13], both reporting good results with plantigrade feet, without pain and with normal ambulation. Yang and Dobbs [16] compared minimally invasive surgery and extensive soft tissue procedures for CVT treatment in both idiopathic and nonidiopathic cases with a mean follow-up of seven years. These authors observed better ankle range of motion and pain scores in those treated with minimally invasive surgery, better preservation of the mobility and function of the patient’s foot.

Regarding nonoperative treatment, the authors generally agree that treatment with serial casts does not resolve this deformity. This may be the reason why the studies carried out in children with CVT treated conservatively are not as abundant as the research of surgical procedures. What is clear is that the sooner treatment begins, the better results will be obtained. It has previously been suggested that CVT treatment should be initiated within the first two weeks after birth or immediately after confirmation of the diagnosis [1]. Becker-Andersen and Reimann reported excellent results in five children whose treatment, without surgery, began just after birth [20]. However, other authors maintain that very few patients obtain a satisfactory correction without surgical treatment. Jacobsen and Crawford presented 11 patients (17 affected feet) with vertical talus, who were treated with serial casts and manipulations [20]. If the correction was not satisfactory, they were treated by surgery. The percentages of relapse after surgical treatment have been reported to be between 18 and 47%, depending on the surgical method used [1].

In the present study, 12 feet (26.1%) had normal TAMBA at the end of the follow-up, and the rest of the feet (with a shorter follow-up) went from having a vertical talus to an oblique talus. The TCA was normal in 97.8% of the feet. Regarding motion, normal dorsal and plantar flexion of the ankle was achieved, resulting in a plantigrade foot without pain. Our results showed the importance of starting treatment within the first seven days after birth. Furthermore, ankle plantar flexion was a good predictor of the need for surgery for total foot correction, establishing that children whose ankle plantar flexion was less than 36° were not operated, and those with more than 40° needed surgery. This may be useful in clinical practice to make prognoses about the evolution of treatment with the conservative method described in the present study.

This study has certain limitations, for example, heterogeneity of the duration of treatment, because it was interrupted when clinical correction of the deformity was achieved, and this was not always of the same duration. The follow-up reported has also been heterogeneous; Since CVT is a rare deformity, our objective was to include all newborns with this deformity in the same hospital, since the described treatment method started to be applied to the last measurement performed in 2021. That is the reason for such heterogeneity.

In summary, the proposed treatment method consisting of daily manipulation and corrective bandages for CVT in newborns avoid surgery in 23 patients (32 feet). Starting this conservative treatment within the first week of life and having a plantar flexion of the ankle lower than 36° were related to the success of the treatment. Knowing these two variables could help determine the need for future surgical treatment.

## Data Availability

The authors have their database available in case it is necessary.

## Code availability

N/A.
